# The burden of myasthenia gravis – highlighting the impact on family planning and the role of social support

**DOI:** 10.3389/fneur.2023.1307627

**Published:** 2023-12-14

**Authors:** Maike Stein, Ulrike Grittner, Regina Stegherr, Lea Gerischer, Frauke Stascheit, Sarah Hoffmann, Meret Herdick, David Legg, Derin Marbin, Andreas Meisel, Sophie Lehnerer

**Affiliations:** ^1^Charité - Universitätsmedizin Berlin, Corporate Member of Freie Universität Berlin and Humboldt-Universität zu Berlin, Department of Neurology with Experimental Neurology, Berlin, Germany; ^2^Charité - Universitätsmedizin Berlin, Corporate Member of Freie Universität Berlin and Humboldt-Universität zu Berlin, Department of Neurology with Experimental Neurology, NeuroScience Clinical Research Center, Berlin, Germany; ^3^Center for Stroke Research Berlin, Charité - Universitätsmedizin Berlin, Berlin, Germany; ^4^Berlin Institute of Health at Charité - Universitätsmedizin Berlin, Digital Health Center, Berlin, Germany; ^5^Charité - Universitätsmedizin Berlin, Corporate Member of Freie Universität Berlin and Humboldt-Universität zu Berlin, Institute of Biometry and Clinical Epidemiology, Berlin, Germany; ^6^Department of Psychiatry of University Hospital Charité in St. Hedwig Hospital Berlin, Berlin, Germany; ^7^Charité - Universitätsmedizin Berlin, Corporate Member of Freie Universität Berlin and Humboldt-Universität zu Berlin, Department of Psychiatry and Neurosciences, Berlin, Germany

**Keywords:** myasthenia gravis, myasthenia, social support, family planning, pregnancy, miscarriage, burden of disease, caregiver

## Abstract

**Background:**

Myasthenia gravis (MG) is a rare autoimmune disease and chronic condition that necessitates specialized care. Patients experience a significant burden of disease affecting various aspects of their lives. The aim of this study was to investigate the impact of MG on family planning, challenges associated with pregnancy, childcare responsibilities and the extent to which MG patients perceive and utilize social support.

**Methods:**

This analysis used data from our main data of a large cross-sectional study built on a questionnaire-based survey encompassing 1,660 MG patients and members of the German Myasthenia Association (Deutsche Myasthenie Gesellschaft), and focused on sociodemographic, clinical and family planning relevant data points.

**Results:**

Decisions regarding family planning were significantly impacted for individuals with MG when MG symptoms started either before or during their family planning (men: *n* = 19 and 29.7%; women: *n* = 156 and 58.4%). In this subgroup a substantial proportion opted against parenthood due to MG (men: *n* = 8 and 50.0%; women: *n* = 54 and 38.0% and/or another *n* = 12 and 8.4% of female participants encountered partner-related refusals). In the subgroup of female SP with MG starting before or during family planning who have reported ever been pregnant the self-reported miscarriage rate was 29.0% (*n* = 51). MG patients with medium incomes or moderate disease severity reported lower levels of perceived social support. 42.7% (*n* = 606) of participants needed assistance in negotiations with health insurers and 28.0% (*n* = 459) needed support for transportation to medical appointments.

**Conclusion:**

This study shows a significant impact of MG on family planning decisions, affecting both women and men, and often resulting in life-altering decisions such as voluntary childlessness due to MG. The significance of social support becomes evident as a vital factor, especially when navigating through the healthcare system. Tailored healthcare approaches, organized guidance and comprehensive support is needed to enable informed decision-making and offer assistance for MG patients.

**Clinical trial registration:**

https://clinicaltrials.gov/study/NCT03979521, Registered 7 June 2019 (retrospectively registered).

## 1 Introduction

Myasthenia gravis (MG) is a rare autoimmune disease with a prevalence of 15–20/100.000 inhabitants (1). Specific antibodies affect the neuromuscular junction and lead to fluctuating fatigability and weakness of the ocular, bulbar and skeletal muscles and can even lead to potentially life-threatening crises. MG is a chronic condition, with the majority of patients needing long-term and often life-long therapy (2). In Germany, specialized care for MG patients is concentrated within 19 integrated Myasthenia Gravis Centers (IMZ) certified by the German Myasthenia Association (Deutsche Myasthenie Gesellschaft, DMG) (3). Complementing medical services, the DMG offers extensive informational and networking resources for patients, caregivers, and healthcare providers.

There is a twofold frequency distribution in the occurrence of the disease, predominantly affecting young women under the age of 40, potentially intersecting with their fertility and family planning stages (4, 5). The decision to start a family is often one of the most important decisions individuals can make in their lives with huge consequences for the individuals social and economic outlook. For individuals with chronic conditions, this decision is often even weightier as they must consider the potential toll on their own health, that of any prospective child, personal relationships and economic activity (6). Therefore, addressing topics such as pregnancy and family planning is a recurring challenge in the medical management of MG patients, affecting both women and men. This phase becomes especially paramount, not only due to potential adjustments in medications but also because the unpredictable course of the disease and can evoke heightened uncertainties. Moreover, social support structures wield a profound influence across personal and professional domains and can serve as a pivotal influencing factor in the decision-making process and psychological well-being. This sub-analysis utilized data from our main dataset (7) to illuminate the challenges encountered in the realm of family planning and the experienced social support among MG patients. Research in the field of family planning and MG has been limited thus far. To the best of our knowledge, the dataset analyzed in this subanalysis represents the most extensive study in this field to date and incorporating sex-related distinctions within this context. This sub-analysis aims to investigate the impact of MG on decisions related to family planning, pregnancy, and parenthood, both in men and women with MG.

## 2 Materials and methods

### 2.1 Standard protocol approvals, registrations, and patient consent

This study received ethics approval by the ethics committee at Charité – Universitätsmedizin Berlin (no. EA1/008/19). No written informed consent was obtained from the study participants since the data collection was completely anonymous. The study was conducted in accordance with the declaration of Helsinki and registered on clinicaltrials.gov (NCT03979521). The STROBE reporting guidelines for observational studies have been applied (8).

### 2.2 Study design and setting

This is a sub-analysis of a cross-sectional questionnaire-based study that was sent out in May 2019 to the 3,262 members of the DMG (7). The study participants (SP) received the study information with the questionnaire as well as a pre-stamped envelope addressed to the coordinating study center. SP were instructed to return their completed questionnaire without any further identifying information to ensure the anonymity of the survey. No refund was given. Returned questionnaires were accepted within the cut-off date of 31^th^ July 2019.

### 2.3 Data sources

The questionnaire contained items on several sociodemographic and disease related dimensions, described in detail in our former publication (7). For this sub-analysis relevant data was: sex, age, income, education, living in partnership, size of family, number of children <14 years old, disease severity, clinical subtype, age at symptom onset and onset regarding time of family planning as well as questions regarding negotiations with health insurance companies and form of transport to treating physician. All questions used for this sub-analysis were asked with a checkbox option, always specified to be answered as a single or multiple-choice option. The questionnaires were scanned and processed with the software TeleForm (OpenText), version 10.9.1.

### 2.4 Standardized scores

To further assess the burden of disease, standardized scores were integrated in the questionnaire and used in this sub-analysis: MG-QoL15 (Myasthenia gravis quality of life, i.e., MG specific HRQoL) (9), MG-ADL (Myasthenia gravis activities of daily living profile) (10), ESSI-D (ENRICHD Social Support Inventory (11, 12) and HADS-D (Hospital anxiety and depression scale) (13–15). The perceived social support was surveyed with the ESSI-D. Low social support was defined by 18 points or less. In the ESSI-D (5-25-point scale), the higher the score, the better is the patients´ situation. Whereas in the MG-QoL15 (0-60-point scale), the MG-ADL (0-24-point scale) and the HADS-D (0-21-point scale for each sub scale anxiety and depression) a high score indicates a worse situation. In the ESSI-D low social support is defined as a sum score of 18 or less and at least two items with 3 or less points (11).

### 2.5 Sociodemographic variables

Educational status was graded into three groups (low, medium, high) on the basis of information on the highest level of education according to the CASMIN classification (16). Information of net household income was based on four income categories in the questionnaire but transformed into three income groups (low = up to 1,188 euro, medium = 1,189–1833 euro, high = 1834 euro and more) to make it comparable with other data sets (17) performed in our prior publication (7).

### 2.6 Statistical analysis

The statistical calculations were performed using IBM Corp. Released 2017. IBM SPSS Statistics for Windows, Version 25.0. Armonk, NY: IBM Corp. using IBM SPSS Statistics 25 and R (version 3.5.3) software (18). Depending on the scale and distribution of the outcome variables, appropriate descriptive statistics (mean, standard deviation, median, interquartile range, absolute and relative frequencies) are presented. Furthermore, parametric and non-parametric statistical tests were used to test for group differences. As effect size measure for Mann–Whitney tests we additionally calculated the probability of a higher value in the group with higher values compared to the other group by calculating the relative frequency of larger values in the first group compared to the other group for all possible pairings (19). A two-sided significance level of α = 0.05 was used. No adjustment for multiple testing was applied in this exploratory study.

## 3 Results

Of the 3,262 contacted members of the DMG, 103 persons were excluded retrospectively from response analysis, because they did not meet the inclusion criteria (e.g., diagnosis of Lambert-Eaton-Myasthenic-Syndrome). The overall response rate was 52.5% (*n* = 1,660). Detailed patient characteristics were previously published (7). Several patient characteristics derived from our main paper’s data, featuring key variables relevant to this sub-analysis, have been included in the supplement of this sub-analysis (Supplementary Table 1). The ESSI-D median sum score was 22 (IQR 19–25), with women perceiving less social support compared to men [Median 21 (IQR 18–24) vs. 23 (IQR 20–25), *p* < 0.001] with the probability of a man having higher values than a woman being 61.1% (Table 1). Low social support was applicable to 22.7% of SP (*n* = 343/1509 with 151 missings in ESSI-D) as shown in the main results (7). The age of the patients (>45 years old or < 45 years old) had no significant influence on the experienced social support. Income and education level had an influence on the reported social support with lower score values in the medium income as well as education group (Median 21 (IQR17-24) and 22 (19–24). SP living together with a partner showed higher scores in the ESSI-D score highlighting higher social support [Median 23 (IQR 20–25) vs. 19 (15–23), *p* < 0.001]. SP with mild or high disease severity or an ocular or bulbar clinical subtype showed higher perceived social support compared to SP with moderate disease severity or clinical subtype of limb muscles affected (Table 1).

**Table 1:** Patient characteristics and their association with social support (ESSI-D, ENRICHD social support inventory, German version).

	*n* (missings*)	median (IQR)	*p*, effect size
ESSI-D sum score (max. 25 points)	1,509 (151)	22 (19–25)	
Gender			
Men	641 (84)	23 (20–25)	<0.001 / 61.1%^a^
Women	865 (66)	21 (18–24)	
Age			
≤45 years	175 (1)	22 (18–24)	0.222 / 52.8%^a^
>45 years	1,325 (148)	22 (19–25)	
Income			
Low	378 (42)	22 (18–24)	<0.001 / 0.04^b^
Medium	264 (19)	21 (17–24)	
High	681 (53)	23 (20–25)	
Education			
Low	381 (71)	23 (19–25)	0.029 / 0.003^b^
Medium	668 (39)	22 (19–24)	
High	443 (38)	23 (20–25)	
Living with a partner			
Yes	1,184 (115)	23 (20–25)	<0.001 / 69.7%^a^
No	308 (32)	19 (15–23)	
Disease severity			
Mild	669 (64)	23 (20–25)	<0.001 / 0.02^b^
Medium	669 (59)	21 (18–24)	
Severe	145 (16)	22 (19–25)	
Clinical subtype (self-reported)			
Ocular	312 (33)	23 (20–25)	<0.001 / 0.01^b^
Generalized, mainly limb muscles affected	612 (42)	21 (18–24)	
Generalized, mainly bulbar muscles affected	424 (49)	22 (19–25)	

a: p value from Mann Whitney U Test, effect size: Probability of higher value in group with larger values compared to group with lower values. b: p value from Kruskal Wallis Test, effect size: η2[H]. *Missings in the second column corresponds to the number of SPs of the categories of the first column who did not answer the questions regarding the ESSI-D sum score.

Regarding both the HADS-D and the MG-QoL15, the SP with perceived low social support (defined by 18 points or less) had higher score values, with probabilities of higher scores of 71.2% in HADS-D and 67.1% in MG-QoL15 (Table 2). This indicates a higher probability for depression and/or anxiety and worse quality of life in the group with perceived low social support. The median MG-ADL score was higher for SP with low social support than for patients with an ESSI-D score > 18 points (probability of higher value was 64.6%) indicating higher disease activity in SP who report low social support (Table 2).

**Table 2:** Perceived social support (ESSI-D) in association with MG-ADL (Myasthenia gravis activities of daily living profile), HADS (Hospital anxiety and depression scale), MG-QoL15 (Myasthenia gravis quality of life).

	All	Low social support (ESSI-D ≤ 18 points)	Medium or high social support (ESSI-D > 18 points)	p (MWU), effect size (Probability of higher value in group with larger ESSI-D values)
MG-ADL (Median (IQR))	4 (1–6)	5 (3–8)	3 (1–6)	<0.001/64.6%
*n* valid (57 missings)	1,452	334	1,118	
HADS-D (Median (IQR))	10 (5–17)	16 (10–22)	9 (4–14)	<0.001/71.2%
*n* valid (62 missings)	1,447	337	1,110	
MG-QoL15 (Median (IQR))	12 (4–25)	21 (10–34)	10 (3–22)	<0.001/67.1%
*n* valid (370 missings)	1,139	246	893	

MWU, Mann Whitney U Test.

If negotiations with health care payers, e.g., in case of requests for medical aids and remedies were necessary, 814 (57.3%) SP stated that they conducted the negotiations themselves without any help (*n* = 1,420 valid answers, *n* = 42 missing, *n* = 198 not applicable). In 269 (18.9%) SP needed additional help from relatives or friends, in 121 (8.5%) SP needed help from a medical doctor. In 55 (3.9%) SP the negotiations were performed by medical doctors solely (Table 3).

**Table 3:** Negotiations with health care payers and form of transport to treating physician.

Who conducts the negotiations with payers (health insurance companies) (*n* = 1,420 valid answers, *n* = 42 missing, *n* = 198 not applicable*)	*n*	%
Myself without any help	814	57.3
Myself with help of relatives, friends	269	18.9
Myself with help of medical doctor	121	8.5
Myself with help of relatives, friends, medical doctor	71	5.0
Medical doctor	55	3.9
Myself with help of relatives, friends, legal advisor / social service, medical doctor	25	1.8
Myself with help of social service / legal advisor	24	1.7
Relatives, medical doctor, social service	17	1.2
Other combinations	24	1.7
Form of transport to treating physician (*n* = 1,636 valid answers, *n* = 24 missing)	*n*	%
Car (driving myself)	811	49.6
Car (driven by someone else)	390	23.8
Public transport	281	17.2
Walking	65	4.0
Taxi	53	3.2
several transport options	20	1.2
Ambulance	16	1.0

*There was an upstream question as to whether remedies or aids had been applied for. The population was only included here if the answer to this question was “yes”.

Help is also required getting to the treating physician in 390 (23.8%) SP, i.e., driven in a car by “someone else.” However, there is a lack of further specification regarding the identity of the individual providing this assistance, such as whether it is a family member or a friend.53 (3.2%) SP stated to take a taxi or ambulance (*n* = 16, 1.0%) to get to their treating physician (Table 3).

1352/1626 (34 missings) (82.3%) SP are living together with one or more persons in the household. At the time the survey was performed 123/1605 (55 missings) (7.7%) SP were living together with a child below 14 years old (Table 4). Most of them were families with one or two children. The SP herself/himself alone (41.3%), the SP with the partner (*n* = 38.8%) or the partner alone (*n* = 6.6%) were mainly responsible for the childcare. Problems in taking care of the children were reported by 16/120 (3 missings) (13.3%) SP (Table 4).

**Table 4:** Persons in MG household and child’s care.

Persons living in patient’s household (incl. patient) (Total *n* = 1,660, missing *n* = 17)	*n*	%
1 person (= MG patient living alone)	291	17.7
2 persons	1,048	63.8
3 persons	162	9.9
4 persons	105	6.4
5 or more persons	37	2.1
Children < 14 years old living in the patient’s household (Total *n* = 1,660, missing *n* = 55)	*n*	%
Total	123	7.7
Number of children < 14 years old living in the household (Total *n* = 123, missing *n* = 4)	*n*	%
1 child	70	58.8
2 children	39	32.8
3 or 4 children	10	8.4
The following questions were only for those with children < 14y		
Mainly responsible for care of children < 14 years (Total *n* = 123, missing *n* = 2)	*n*	%
MG patient her/himself alone	50	41.3
MG patient & partner	47	38.8
partner alone	8	6.6
MG patient her/himself & partner & relatives	7	5.8
MG patient her/himself & partner & domestic help	2	1.7
MG patient her/himself & domestic help	2	1.7
other combinations	5	4.1
Problems in care of children < 14 years (Total *n* = 123, missing *n* = 3)	*n*	%
Yes	16	13.3

All SP were asked if MG symptoms started before, during or after the period of family planning (Table 5). 34.8% (*n* = 273/783, 148 missings) of women reported that the MG symptoms started before or during the period of family planning. In female SP who developed first symptoms of MG before or during family planning, 58.4% (156/267, 6 missings) stated that the disease had influenced family planning (Table 6). Nearly half of all SP (47.2%, 102/216, 25 missings) reporting that MG influenced family planning renounced to have children at all. More than one third, 34.3%, (74/216) changed their own life planning and decided to get pregnant at a later point in time. 5.6% (12/216) stated that the partner refused to have children due to MG (Table 6).

**Table 5:** Myasthenia gravis (MG) onset in relation to family planning.

	All patients (*n* = 1,387, missing *n* = 273)	Female patients (*n* = 783, missing *n* = 148)	Male patients (*n* = 601, missing *n* = 124)
MG started before family planning, *n* (%)	249 (18.0)	207 (26.4)	42 (7.0)
MG-ADL Median (IQR)	3 (1–6), missing *n* = 11	3 (1–6), missing *n* = 10	3 (2–5), missing *n* = 1
MG started during family planning, *n* (%)	91 (6.6)	66 (8.4)	25 (4.2)
MG-ADL Median (IQR)	4 (2–7), missing *n* = 6	4 (2–8), missing *n* = 5	4 (2–6), missing *n* = 1
MG started after family planning, *n* (%)	1,047 (75.5)	510 (65.1)	534 (88.9)
MG-ADL Median (IQR)	4 (1–6), missing *n* = 43	5 (2–8), missing *n* = 22	3 (1–5), missing *n* = 21

**Table 6:** Myasthenia gravis (MG) effect on family planning.

	All study participants (SP) (*n* = 1,438, missing *n* = 222)	Female SP (*n* = 793, missing *n* = 138)	Male SP (*n* = 642, missing *n* = 83)	Female SP with MG starting before or during period of family planning (*n* = 267, missing *n* = 6)	Male SP with MG starting before or during period of family planning (*n* = 64, missing *n* = 3)
MG affecting family planning, *n* (%)	241 (16.8)	200 (25.2)	41 (6.4)	156 (58.4)	19 (29.7)
Age Median (IQR)	66 (54–76) missing *n* = 7	58 (49–71) missing *n* = 1	72 (64–78) missing *n* = 3	49 (39–58) missing *n* = 1	59 (46–68) missing *n* = 0
If Yes	*n* = 216, missing *n* = 25	*n* = 181, missing *n* = 19	*n* = 35, missing *n* = 6	*n* = 142, missing *n* = 14	*n* = 19, missing *n* = 3
I renounced children	102 (47.2)	81 (44.8)	21 (60.0)	54 (38.0)	8 (50.0)
My partner refused to have children	12 (5.6)	10 (5.5)	2 (5.7)	7 (4.9)	--
I renounced children & my partner refused to have children	7 (3.2)	7 (3.9)	--	5 (3.5)	--
We have decided on a later pregnancy	74 (34.3)	65 (7.0)	9 (25.7)	61 (43.0)	6 (37.5)
We have decided on an earlier pregnancy	21 (9.7)	18 (9.9)	3 (8.6)	15 (10.6)	2 (12.5)

In difference to women, 11.2% (*n* = 67/601, 124 missings) of men reported that the MG symptoms started before or during the period of family planning (Table 5). In male SP who developed MG symptoms before or during family planning, 29.7% (19/64, 3 missings) said the disease had influenced family planning (Table 6). The median MG-ADL score was 3 points in the group where MG started before family planning, and 4 points in the group where MG started during family planning. However, the self-reported MG-ADL relates to the time when the SP completed the questionnaire, irrespective of the timing of family planning. Overall, 77.4% (697/901, 30 missings) of all female SP stated that they had been pregnant in the past (Table 7); 27.0% (182/673, 24 missings) of them stated that they had had a miscarriage in the past. Of the female SP who received the diagnosis after family planning, 89.2% (445/499, 11 missings) had already been pregnant in the past, whereas 66.1% (179/271, 2 missings) of all female SP diagnosed with MG before or during family planning reported former pregnancy (Table 7).

**Table 7:** Pregnancy and miscarriages in female myasthenia gravis (MG) patients (SP, study participant).

	All female patients (*n* = 901, missing *n* = 30)	Female SP with MG starting after period of family planning (*n* = 499, missing *n* = 11)	Female SP with MG starting before or during period of family planning (*n* = 271, missing *n* = 2)
Ever been pregnant, *n* (%)	697 (77.4)	445 (89.2)	179 (66.1)
If pregnant in the past: Experience of miscarriage (at least one)	All female patients (*n* = 673, missing *n* = 24)	Female SP with MG starting after period of family planning (*n* = 426, missing *n* = 19)	Female SP with MG starting before or during period of family planning (*n* = 176, missing *n* = 3)
*n* (%)	182 (27.0)	115 (27.0)	51 (29.0)

## 4 Discussion

The aim of this study was to investigate the impact of MG on family planning decisions, pregnancy and parenthood and the extent to which MG patients perceive and utilize social support among a cohort of 1,660 MG patients and members of the DMG.

### 4.1 Family planning and pregnancy

Our results revealed a higher reported incidence of MG symptoms among women (34.8%) compared to men (11.2%) occurring during family planning which aligns with the recognized peak of the disease in young women (1). Notably, a considerable proportion of both women (58.4%) and men (29.7%) indicated that MG had influenced their decisions regarding family planning within this subgroup, corroborating findings from previous research (20). Accordingly, the number was similar for MG (52.9%) in a study conducted by Alanazy et al. (21). The repercussions of MG on family planning decisions are consequential, prompting certain patients to make substantial choices. Our results showed that 38.0% of female participants with MG starting before or during period of family planning opted against having children and/or another 8.4% of female participants faced partner-related refusals. An even more pronounced decision was observed among men, with 50.0% choosing not to have children. The study conducted by Ohlraun et al. showed similar results regarding MG and demonstrated that common concerns among female MG patients refusing children encompassed potential effects of MG medication on the unborn child (87.1%), exacerbation of MG during pregnancy (70.3%) and fear of the delivery process (64.0%) – factors that resonate with considerations in other chronic neurological disorders (22, 23). Regarding these concerns, particularly during the first trimester and the postpartum period the risk for exacerbation of MG symptoms is increased by up to 30% (24), highlighting the importance of stabilizing myasthenic symptoms before pregnancy (25–27). A short disease duration and advanced clinical severity were associated with a profound probability for MG worsening (27–29). But fortunately there are several therapeutic options available which are considered safe during pregnancy which include pyridostigmine, prednisolone, and azathioprine and rescue therapies with intravenous immunoglobulin (30). However, the occurrence of preterm rupture of amniotic membranes was more pronounced, particularly in cases where MG deteriorated during pregnancy (25, 31), other challenging conditions include fetal acetylcholine receptor antibody-associated disorders (FARAD) (32) and around 10% of newborns may develop transient neonatal myasthenia due to antibodies crossing the placenta (33–35). Other studies reported that neonates of women with MG were more likely of being born prematurely (36). However, there is also conflicting evidence from a large population-based cohort study that found no adverse pregnancy outcomes in MG patients and their newborns, including having congenital malformation or decreased APGAR score (37). A systematic review highlights the importance of timing and safety of thymectomy, as it has been observed that the incidence of neonatal MG was much lower if the mother had thymectomy before delivery (25), and there is also reduced tendency for myasthenic exacerbation during pregnancy (38). In our study the self-reported miscarriage rate was 29.0% among female SP with MG symptoms starting before or during family planning. Previous studies have reported slightly lower miscarriage rates ranging from 15–20% in MG, similar to the miscarriage rate in the general population of 10–20% (23, 39–41). It is important to note that precise data remains challenging to ascertain due to limited sample sizes and potential selection biases in reports and data does not allow causal inference. Nevertheless, previous research has indicated that psychological distress is frequently observed following pregnancy loss (42). Consequently, individuals with MG not only face the physical challenges associated with their condition during pregnancy and motherhood but also necessitate a special focus on psychological support within this context. In this context, it is crucial for treating physicians to provide effective counseling and engage in proactive communication while fostering a safe and supportive environment to sensitively address family planning issues, aiming to inform patients thoroughly and collaboratively determine the best individual solution, to prevent voluntary childlessness in MG.

### 4.2 Social support and its multifaceted impact on MG patients

Social support structures also play a crucial role in managing childcare responsibilities. However, 41% of SP reported handling childcare alone. 7.4% of SP are living with children, and within this subgroup a higher proportion having only one child compared to having two or more children. One contributing factor could be potential concerns about the ability to care for a child, as this has been expressed by 70.5% of female MG patients in the study by Ohlraun et al. (23). This emphasizes the need for robust social support networks also in the context of family planning. However, our main study showed that 20% of all SP reported low social support (7), and that these affected SP experienced heightened difficulties in daily activities, increased anxiety and depression symptoms, lower quality of life (measured by instruments such as MG-ADL, HADS-D, and MG-QoL15r). Partnerships enhance perceived social support. Other studies have reported even more pronounced deficits with up to 50% of MG patients indicating reduced ‘social positivity’ or non-satisfactory social support (42, 43). However, considering MG’s unique demands (44), there is a high need for socio-legal guidance among MG patients (45). Our findings further demonstrated this need with 42.7% SP required assistance in negotiations with health insurers. Furthermore, nearly a third of SP (28.0%) needed transportation assistance to medical appointments (e.g., driven by someone else, taxi, ambulance), both depending on the availability of social support structures. Although a significant percentage of MG patients are autonomous regarding commuting to medical appointments, the majority of those requiring transport assistance primarily rely on support from others (driven a car by someone else). These are most likely partners, relatives, or individuals within their personal network. This necessitates an adjustment of the patient’s environment to meet their needs. While causality remains uncertain, the association hints at intricate relationships between social support, disease manifestation, and psychological well-being. Interestingly, our results revealed that SP with medium incomes received less social support than those with lower and higher incomes. This phenomenon may stem from the fact that households and individuals with higher incomes might have more resources and access to a broader network of supportive measures, including professional networks and colleagues. Conversely, lower-income individuals might rely on social connections and community support to a greater extent. The literature exploring the individual income level in the context of neurological diseases like MG is relatively scarce. However, social support is undeniably crucial for individuals with neurological diseases but its influence extends beyond just income and may be impelled by various other social determinants, including education, occupation, and ethnicity (46).

Our study’s limitations include concerns about representativeness, given that the majority of participants were affiliated with a national patient organization (DMG). Demographic differences raise potential concerns about the generalizability of findings and selection bias is a possibility. Additionally, due to the cross-sectional design, causality cannot be inferred. However, notably, our study, encompassing 1,660 participants, is the largest on this topic so far, with sex distribution mirroring outpatient clinic data and relevant literature. The study’s strengths include a representative cohort and substantial participant pool (*n* = 1,660, response rate 52.5%), yielding a robust real-world dataset. Nevertheless, bias among non-responders cannot be ruled out, as the responses of this group might have diverged from those of the responders if they had participated.

Our results reveal a substantial impact of MG on individuals, both women and men, within the realms of family planning. This influence can lead to far-reaching decisions with voluntary childlessness due to MG. Furthermore, the pivotal role of social support emerges as a crucial factor, especially when navigating the healthcare system. Addressing these multifaceted challenges on individuals with MG highlights the necessity for tailored healthcare approaches, structured guidance, and comprehensive support to facilitate informed decision-making and provide assistance. Our findings establish a basis for further prospective studies on family planning and dynamics of social support among MG patients.

## Data availability statement

The raw data supporting the conclusions of this article will be made available by the authors, without undue reservation.

## Ethics statement

The studies involving humans were approved by Charité Universitätsmedizin Berlin. The studies were conducted in accordance with the local legislation and institutional requirements. Written informed consent for participation was not required from the participants or the participants’ legal guardians/next of kin because No written informed consent was obtained from the study participants since the data collection was completely anonymous.

## Author contributions

MS: Writing – original draft. UG: Formal analysis, Writing – review & editing. RS: Formal analysis, Writing – review & editing. LG: Writing – review & editing. FS: Writing – review & editing. SH: Writing – review & editing. MH: Writing – review & editing. DL: Writing – review & editing. DM: Writing – review & editing. AM: Conceptualization, Resources, Supervision, Writing – review & editing. SL: Conceptualization, Formal analysis, Writing – original draft, Investigation.
