# Supplementary material for: The burden of myasthenia gravis – highlighting the impact on family planning and the role of social support
Source: Front Neurol. 2023 Dec 14;14:1307627. doi: 10.3389/fneur.2023.1307627 (PMC10755901; doi:10.3389/fneur.2023.1307627)
Supplement: Supplementary file 1 [file Data_Sheet_1.PDF]

Supplementary Material

The burden of myasthenia gravis - highlighting the impact on family planning and the role of social support

Maike Stein*, Ulrike Grittner, Regina Stegherr, Lea Gerischer, Frauke Stascheit, Sarah Hoffmann, Meret Herdick, David Legg, Derin Marbin, Andreas Meisel, Sophie Lehnerer

* Correspondence:
Dr. Maike Stein, Charité – Universitätsmedizin Berlin, Center for Stroke Research Berlin, Charitéplatz 1, 10117 Berlin, Germany, Email: maike.stein@charite.de

Table1 Clinical and sociodemographic characteristics of study participants as published in Lehnerer et al. (2021), MG=Myasthenia gravis

**Disease severity** (54 missing)	**n**	**%**
Low	733	45.7
Women	369	41.1
Men	362	51.4
Medium	728	45.3
Women	440	49.0
Men	287	40.8
High	145	9.0
Women	89	9.9
Men	55	7.8
**Clinical subtype** (41 missing)	**n**	**%**
Ocular	345	21.3
Generalized	1127	69.6
mainly limb muscles affected	654	48.0
mainly bulbar muscles affected	473	42.0
„I do not know“	147	9.1
**Marital status** (16 missing)	**n**	**%**
Married, living together with the partner	1170	71.2
Married, living separate from the partner*	29	1.8
Single*	157	9.5
Widowed*	171	10.4
Divorced*	117	7.1
* Of which living in partnership (8 missing)	126	27.0
**Living in partnership (married or not married)**	**1296**	**85.8**
**MG was cause of separation** (in case of separation or divorce)	**n**	**%**
MG was no cause of separation	127	68.2
MG was of minor importance	6	3.8
MG was of medium importance	22	14.0
MG was of high importance	22	14.0
**MG affecting family planning** (222 missing)	**n**	**%**
Yes	241	16.8
**Net household Income (unweighted)** (227 missing)	**n**	**%**
< 1000€	62	4.3
1000€ - 2499€	525	36.6
2500€ - 5000€	664	46.3
> 5000€	182	12.7
**Being afraid of old age poverty** (27 missing)	**n**	**%**
Yes	486	29.8
…this is due to MG (7 missing)	320	66.8
**Age at onset of symptoms** (123 missing)	**Mean (SD)**	

Total (n=1537)	49.3 (19.7)
Men (n=683)	58.6 (15.3)
Women (n=850)	41.8 (19.6)
**Duration from first symptoms to diagnosis (years)** (157 missing)	**Mean (SD)**
Total (n=1503)	2.14 (5.8)
Men (n=670)	1.28 (4.8)
Women (n=829)	2.83 (6.4)
**Disease Duration (since diagnosis)** (45 missing)	**Mean (SD)**
Total (n=1615)	13.6 (11.6)
Men (n=710)	11.0 (9.4)
Women (n=905)	15.6 (12.7)

Table 2: Overview MG-ADL, MG-QoL15, HADS, CFQ, ESSI-D and subgroups as published in Lehnerer et al. (2021)

**Parameter**	**All**	**Men**	**Women**	**gMG**	**oMG**	**Refractory MG**	**Ach-R-Abs pos MG**	**Musk-Abs pos MG**	**Thym-ectomy**	**EOMG**	**LOMG**
**n** (missing)	1660 (0)	725 (4)	931 (4)	1127 (41)	345 (41)	228 (200)	837 (41)	82 (41)	743 (43)	176 (11)	1473 (11)
%	100	43.8	56.2	69.6	21.3	15.6	51.7	5.1	45.9	10.7	89.3
**MG-ADL**	4	3	4	4	2	7	3	5	3	3	4
Median (IQR)	(1/6)	(1/5)	(7/12)	(2/7)	(1/4)	(4/10)	(1/6)	(2.25/7)	(1/6)	(1/6)	(2/6)
p-values	-	**<0.00**		**<0.00**	**<0.00**	**<0.00**	**<0.00**	**0.009**	**0.047**	0.292	
**MG-QoL15**	12	9	15	16	6	29	11	16	11	12	12
Median (IQR)	(4/25)	(3/21)	(5/28)	(5/28)	(2/14)	(19/38)	(3/23)	(8/25)	(3/24)	(3/28)	(4/24)
p-values	-	**<0.00**		**<0.00**	**<0.00**	**<0.00**	**<0.00**	0.072	**0.018**	0.523	
**HADS**	10	9	11	11	8	12	9	10	9	10	10
Median (IQR)	(5/17)	(4/15)	(6/18)	(6/18)	(4/13)	(8/19)	(5/15)	(5/18)	(5/17)	(5/17)	(5/17)
p-values	-	**<0.00**		**<0.00**	**<0.00**	**<0.00**	**0.018**	0.313	0.119	0.995	
**HADS-A** ≥ 8 p.	520	179	339	393	78	86	234	26	229	64	452
n (%)	(32.5)	(25.5)	(37.9)	(36.1)	(23.2)	(38.4)	(28.4)	(33.8)	(31.6)	(37.2)	(31.9)
**HADS-D** ≥ 8 p.	446	184	260	347	57	84	191	24	177	40	402
n (%)	(27.9)	(26.1)	(29.2)	(31.9)	(16.8)	(36.8)	(23.4)	(30.8)	(24.5)	(23.1)	(28.4)
**CFQ** sum (Likert)	17	16	17	18	14	21	16	19	16	16	17
Median (IQR)	(12/21)	(12/21)	(12/22)	(13/22)	(11/18)	(17/25)	(12/21)	(13/23)	(12/21)	(12/21)	(12/22)
p-values	-	**0.002**		**<0.00**	**<0.00**	**<0.00**	**<0.00**	**0.029**	**0.002**	0.187	
**CFQ** ≥4 (Binary)	989	426	560	728	161	183	483	55	424	103	880
n (%)	(66.7)	(63.3)	(69.4)	(72.5)	(50.8)	(80.3)	(63.9)	(71.4)	(63.6)	(63.2)	(67.1)
**ESSI-D** ≤ 18 p.	343	102	241	253	55	60	156	9	151	42	300
n (%)	(22.7)	(15.9)	(27.9)	(24.4)	(17.6)	(26.3)	(20.2)	(11.8)	(21.8)	(24.0)	(22.6)
p-values	-	**<0.00**		**0.041**	**0.041**	0.122	**0.045**	**0.043**	0.503	0.687	

Abbreviations *gMG = Generalized myasthenia gravis, oMG = Ocular MG, EOMG = Early Onset Myasthenia Gravis (≤ 45 years old), LOMG = Late onset myasthenia gravis (> 45 years old), MG-Qol15 = Myasthenia gravis quality of life, MG-ADL = Myasthenia gravis activities of daily living profile, CFQ-11 = Chalder Fatigue scale, ESSI-D = ENRICHED Social Support Inventory and HADS-D = Hospital anxiety and depression scale; p-values indicate significance level or between men and women, or EOMG and LOMG or otherwise in the specific domain (e.g. gMG or Thymectomy) yes/no.*
